# Genome-Wide Association Studies of the Human Gut Microbiota

**DOI:** 10.1371/journal.pone.0140301

**Published:** 2015-11-03

**Authors:** Emily R. Davenport, Darren A. Cusanovich, Katelyn Michelini, Luis B. Barreiro, Carole Ober, Yoav Gilad

**Affiliations:** 1 Department of Human Genetics, University of Chicago, Chicago, IL, United States of America; 2 Department of Pediatrics, Saint Justine Hospital Research Centre, Montreal, Canada; University of Illinois, UNITED STATES

## Abstract

The bacterial composition of the human fecal microbiome is influenced by many lifestyle factors, notably diet. It is less clear, however, what role host genetics plays in dictating the composition of bacteria living in the gut. In this study, we examined the association of ~200K host genotypes with the relative abundance of fecal bacterial taxa in a founder population, the Hutterites, during two seasons (n = 91 summer, n = 93 winter, n = 57 individuals collected in both). These individuals live and eat communally, minimizing variation due to environmental exposures, including diet, which could potentially mask small genetic effects. Using a GWAS approach that takes into account the relatedness between subjects, we identified at least 8 bacterial taxa whose abundances were associated with single nucleotide polymorphisms in the host genome in each season (at genome-wide FDR of 20%). For example, we identified an association between a taxon known to affect obesity (genus *Akkermansia*) and a variant near *PLD1*, a gene previously associated with body mass index. Moreover, we replicate a previously reported association from a quantitative trait locus (QTL) mapping study of fecal microbiome abundance in mice (genus *Lactococcus*, rs3747113, *P* = 3.13 x 10^−7^). Finally, based on the significance distribution of the associated microbiome QTLs in our study with respect to chromatin accessibility profiles, we identified tissues in which host genetic variation may be acting to influence bacterial abundance in the gut.

## Introduction

Humans have complex interactions with the bacteria that live in and on their bodies, referred to as the microbiota[1]. Alterations in the microbiota, particularly in the gut, have been linked to variation in risk for obesity[2–4], celiac disease[5], Crohn’s disease[6, 7], ulcerative colitis[8–11], gastroenteritis[12], asthma[13], and inflammatory bowel disease[14, 15]. Therefore, understanding the factors that determine and maintain gut microbiome composition has the potential to unlock therapies to improve human health. Several environmental factors have been shown to play a role in determining gut microbiome composition such as the method of delivery at birth[16], formula vs. breast feeding as an infant[17], and diet[18–21]. One major factor that has yet to be examined in detail is the role of host genetics.

Several groups have investigated the heritability of the gut microbiome in humans and model organisms; however, estimates of genetic contribution to bacterial abundance vary between studies. One early study used temperature gradient gel electrophoresis (TGGE) to examine the similarity of the bacterial 16S rRNA genes from gut bacteria between individuals with varying degrees of relatedness. Their findings showed TGGE profiles were increasingly similar as the relatedness between pairs of individuals also increased[22]. Although this is consistent with a heritable component to the microbiome, genetic effects are confounded by similarity in environment, as related individuals likely shared environments throughout their lives to a greater extent than unrelated individuals. Another study estimated microbiome heritability using 16S rRNA gene sequencing in twin pairs and parent-offspring trios by examining relatedness using a pairwise distance measurement of microbial composition[3]. Although microbiome composition was more similar between twins than between parent-offspring pairs or unrelated individuals, the microbiome of monozygotic twins was not more similar than that of dizygotic twins, arguing against a strong genetic component to microbiome composition. This study was, however, small (~20–30 twin pairs in each category) and only examined broad measures of the microbiome composition rather than individual bacterial abundances. More recently, a study on the heritability of common gut bacteria in >400 twin pairs suggested host genetics plays a role in determining gut microbiome composition in humans, with some bacterial taxa having heritability estimates as high as 0.39[23].

While evidence for host genetics influencing the gut microbiota is gaining traction, we still lack an understanding of what genes or genetic variants in the human genome might potentially influence bacterial profiles. Many studies have focused on candidate genes, where either natural variation segregating in humans[24–26] or gene knockout models in mice[27–29] were associated with differences in the microbiome. However, only one study to date has performed a genome-wide scan for variants associated with bacterial abundance in the gut: Benson *et al.[30]* identified 18 quantitative trait loci (QTL) associated with various bacterial taxa in the gut using advanced intercrossed mouse lines. This study demonstrates the utility of genome-wide approaches, however, no such study has been reported in humans.

To address this gap, we examined the fecal microbiome from the Hutterites, a religious isolate living in North America. Importantly, members of this population live and eat on large communal farms, called colonies, limiting inter-individual variation in environmental exposures that might mask genetic effects on microbiome composition. In particular, meals are prepared and eaten in a communal kitchen and dining room, respectively. Previous work in this population examined temporal differences between winter and summer gut microbiomes[18]. Here, we examined the same individuals for sex, age, and genetic effects on microbiome composition using both the winter data (n = 93) and summer data (n = 91), separately. In addition, we considered a composite microbiome (“seasons combined”), where the relative abundances of bacterial taxa sampled in winter and summer are averaged for any individual where stool was available from both seasons (n = 127; see Materials and Methods). While the abundance of only one bacterial taxa correlated with age (genus *Bifidobacterium*), at least four bacterial taxa in each season were differentially abundant by sex. In addition, we identified at least eight bacterial taxa in each season that are associated with at least one single nucleotide polymorphism (SNP) at a genome-wide significance level. Finally, we identified pathways and candidate tissues where host genetic variation may be acting to influence bacterial abundance in the gut.

## Materials and Methods

### Ethics statement

The protocol was approved by the University of Chicago IRB (protocol 10-416-B). Written informed consent was obtained from all adult participants and the parents of minors. In addition, written assent was obtained from minor participants.

### Accession Numbers

The data for the 16S rRNA amplicon sequencing, genotypes, GWAS results, and metadata have been deposited in dbGaP under accession numbers phs000680 and phs000185.

### Microbial data collection

Stool collection, DNA extraction, 16S rRNA gene sequencing, and classification were performed as described previously[18]. Briefly, stool samples were collected during two seasons (winter and the following summer). Sequences were classified using a Naïve-Bayesian classifier as implemented in mothur[31]. In our previous study of seasonal differences in microbiome composition, individuals were excluded if they took antibiotics within 6 months of either the summer or winter sampling date. For this study, in which seasons were considered individually, samples were excluded if the individual had taken antibiotics within the previous 6 months the sampling date for each within season analysis. Additional samples were excluded for individuals without genotyping data. After exclusions, samples for 93 individuals in winter (60 females and 33 males), 91 individuals in summer (57 females and 34 males), and 127 individuals in the combined sample (79 females and 48 males) remained. For all analyses, sequencing reads were randomly subsampled to a maximum depth of 2 million reads per technical replicate and technical replicates were combined, resulting in approximately 4 million reads per individual per season. Taxon abundances were standardized to the total number of reads subsampled to generate relative abundance measures.

### Genotype data

Genotyping in 1415 Hutterite individuals (including the 127 individuals considered in this study) was performed using either the Affymetrix 500k Array Set, the Genome-Wide Human SNP Array 5.0, or the Genome-Wide Human SNP Array 6.0 as part of a long-term research program studying the genetic basis of complex phenotypes in the Hutterites[32–35]. Genotypes for the Affymetrix 500k array set were called using BRLMM (http://media.affymetrix.com/support/technical/whitepapers/brlmm_whitepaper.pdf) and genotypes for the Genome-Wide Human SNP Array 5.0 and 6.0 were called using Birdseed[36]. SNPs were initially quality filtered using the following criteria across 1415 individuals: minor allele frequency ≥ 5%, call rates ≥ 95%, Hardy-Weinberg equilibrium *P*-values ≥ 0.001, and fewer than 5 Mendelian errors across all individuals. 271,365 SNPs that were on all three platforms remained after quality control filtering and re-annotation to the human genome version 19 (hg19, dbSNP135).

### Bacterial data pre-processing

#### Individual seasons

The following bacterial data processing was done separately for samples collected in the winter and in the summer. Because there would be limited power to detect associations in rare members of the microbiota, we first eliminated from subsequent analyses bacterial taxa that did not have at least one read in at least 75% of individuals (number of bacterial taxa remaining: winter = 7 phyla, 15 classes, 26 orders, 45 families, and 80 genera. summer = 7 phyla, 15 classes, 21 orders, 38 families, and 71 genera). Next, taxon data was fit to a standard normal distribution across individuals by quantile normalization using the R function qqnorm[37]. Finally, to limit the burden of multiple testing, we removed any taxon that was highly correlated (Pearson correlation ≥ 0.9) with either a taxon at a lower taxonomic level (the taxon at the higher level was removed) or at the same level (the taxon listed first alphabetically was removed). The pairs of highly correlated bacterial taxa are shown in S1 Table. The final dataset for winter consisted of 116 bacterial taxa: 3 phyla, 3 classes, 8 orders, 22 families, and 80 genera. The final dataset for summer consisted of 104 bacterial taxa: 3 phyla, 3 classes, 8 orders, 19 families, and 71 genera.

#### Combining seasons, with normalization

Our previous work revealed broad, temporal differences in gut bacterial abundance between winter and summer in this population[18]. However, having the largest possible sample increases power in genetic association studies, which is critical given the number of tests performed. To this end, we normalized bacterial taxon abundance in each season separately before combining data from the two seasons and included only common taxa that had at least one read in 75% of individuals in both seasons (7 phyla, 15 classes, 21 orders, 38 families, and 70 genera). Specifically, within each season, taxon data was fit to a standard normal distribution across individuals by quantile normalization using the R function qqnorm[37]. Normalized bacterial taxon abundances were then averaged for any individuals who were sampled in both seasons. To further limit the burden of multiple testing, we removed bacterial taxa that were highly correlated, as described above. The final dataset consisted of 102 bacterial taxa: 3 phyla, 3 classes, 7 orders, 19 families, and 70 genera. To ensure that seasonal effects were accounted for, principal component analysis was performed on all genera level classifications in the final, normalized data set using prcomp in R; season was not correlated with any of the top 10 principal components in this final dataset (S1 Fig). We refer to this dataset as the “seasons combined” from hereon in.

### Bacterial correlations with age and sex

Correlations of individual bacterial taxa to age and sex were performed using linear mixed models that corrected for the relatedness between individuals, as implemented in GEMMA (v0.94)[38]. First, relevant covariates were regressed out of normalized bacterial abundances (sex and colony/date of collection for examining taxa correlations with age; and age and colony/date of collection for examining taxa correlations with sex). Samples were collected from five colonies on separate days; therefore, colony and date of collection are confounded. With this understanding, we refer to ‘date of collection’ rather than ‘colony/date of collection’ throughout the manuscript. Linear models were run using GEMMA specifying the–notsnp option. Relatedness matrices were calculated using identity by descent from genotype data[39]. Significance was assessed using a likelihood ratio test and multiple testing corrections were done using q-values (S5 and S6 Tables)[40].

### Diversity metrics

All bacterial taxa present at the genus level in each sample were used to calculate alpha diversity metrics (richness, Shannon diversity, and evenness), without quantile normalization applied to relative abundances and no taxa eliminated due to rarity. Diversity metrics were calculated using the vegan package in R[41].

### Chip heritability of gut bacteria

Relevant covariates (age, sex, and date of collection) were regressed from each bacterial taxon within each seasonal analysis. **“**Chip heritability” (or percent variance explained, PVE, S2 Table) was calculated for the residuals of each taxon using GEMMA v0.94[38]. PVE is considered non-zero if the standard error measurements do not intersect zero.

### Bacterial associations to host genetic variation

Prior to association testing, age, sex and date of collection were regressed from normalized bacterial taxon relative abundance data. GEMMA (v0.94) was used to perform GWAS on the residuals, including the relatedness matrices as described above to account for inter-individual relatedness. SNPs were eliminated if their minor allele frequency was lower than 10% in the individuals tested. A total of 211,319 SNPs in the winter analysis, 210,924 SNPs in the summer analysis, and 212,153 SNPs in the “seasons combined” analysis were examined. Both a conservative Bonferroni corrected *P-*value threshold and less conservative q-value thresholds (0.2 and 0.1) were considered to correct for multiple testing within each genome-wide association study[40].

The number of bacterial taxa within each season that had both non-zero “chip heritability” and at least one genome-wide significant association were examined. To determine the significance of this overlap within each season, “chip heritability” estimates were permuted across bacterial taxa and the overlap of taxa that had both non-zero permuted “chip heritability” and at least one genome-wide significant SNP association was calculated, generating a null distribution of the number of overlaps expected by chance. An empirical *P*-value was calculated by dividing the number of overlaps equal to or greater than the observed number of overlaps for that season by the number of permutations (10,000) in the null distribution.

### Functional annotation enrichment

It is unclear in what tissues genetic variation might be acting in the human body to influence bacterial abundance in the gut. To investigate this, for each bacterial taxon that either had a genome-wide significant association through GWAS or non-zero “chip heritability”, we examined the enrichment of GWAS SNPs in DNase hypersensitivity (DHS) peaks for 16 tissues. In the human genome, DHS peaks mark areas of accessible chromatin, which is assumed to typically participate in gene regulatory functions. Many of these accessible regulatory regions of the human genome are cell-type or temporally specific[42]. Therefore, candidate cell-types where genetic variation may be acting to influence an ultimate phenotype can be determined by examining the enrichment of strongly associated GWAS SNPs in open chromatin across tissues. Maurano *et al*.[43] demonstrated the utility of this approach, identifying immune cells as the candidate cell types for Crohn’s Disease and multiple sclerosis and heart cells for QRS duration. In this study, we took this approach to identify candidate tissue types where host genetic variation may be acting to determine bacterial abundance in the gut.

DNase-I hypersensitivity data from Maurano *et al*.[43] was downloaded in bed format from http://www.uwencode.org/proj/Science_Maurano_Humbert_et_al/ on February 12^th^, 2014. The 349 available cell lines were clustered into 16 tissue classifications by calculating Euclidean distances for DNase peaks between all pairs of lines (S3 Table). DHS peaks for these tissues were determined by taking the intersection of all DHS peaks over all cell lines classified as that tissue in the previous step using intersectBed[44]. Overlaps between the genotyped Hutterite SNPs included in this study and each DHS peak for each tissue were determined using intersectBed.

We performed the following analysis for each of our bacterial abundance GWAS where either i) at least one SNP met suggestive genome-wide significance, or ii) there was non-zero “chip heritability” for that taxon (number of taxa examined in winter = 23, summer = 22, “seasons combined” = 18). For increasingly significant *P*-value thresholds, we examined the enrichment of SNPs identified in our GWAS (*P*-values at or below the given threshold) in DHS peaks from each of 16 tissues, compared to the genome-wide distribution of all tested SNPs located within DHS peaks of the same tissue. The enrichment of SNPs in DHS peaks per tissue (called “enrichments” in the remainder of this section) were calculated as described in Maurano *et al*., with the exception that the following *P*-value thresholds were examined for each bacterial abundance GWAS only if at least 50 SNPs had *P*-values at or below the cutoff: 1.0, 0.5, 0.1, 0.05, 0.01, 0.005, 0.001, 0.0005, 0.0001, 0.00005, 0.00001, 0.000005 (S1 File). Significance of enrichment was assessed for each tissue for the smallest *P*-value bin size per bacterial taxa abundance GWAS by comparing the actual tissue enrichment observed to the distribution of tissue enrichments observed in permuted GWAS data. Specifically, normalized bacterial abundances were randomly assigned to individuals 10,000 times for each seasonal analysis. GWAS was performed for each of these permutations and the enrichment of top GWAS associations in DHS peaks was calculated for each tissue in each permutation, generating a null distribution of enrichments per tissue. The number of SNPs falling into the most significant *P*-value bin with at least 50 SNPs varied by GWAS. For each comparison, the number of SNPs in the smallest bin was matched in the permutations to the number of top SNPs in the smallest *P*-value bin from the actual GWAS. For example, if the actual GWAS for taxa “A” contained 63 SNPs in the smallest *P*-value bin, then the top 63 SNPs were chosen to calculate enrichment in each permutation. An empirical *P*-value was calculated by dividing the number of enrichments in the null distribution that were equal to or larger than the actual enrichment value by the total number of permutations (10,000) for each tissue (S4 Table). Note that reported *P*-values are not corrected for multiple testing.

### Gene set enrichment analysis (GSEA)

GSEA was performed for any bacterial abundance GWAS with at least one significantly associated SNP using the R package postgwas[45]. Each tested SNP was assigned to the closest gene using Ensembl release 75 and any SNP further than 10kb from a gene was eliminated from analysis. An aggregate gene-wise *P*-value was calculated using the function gene2p (method = SpD) in postgwas by taking into account the dependency structure between SNPs. Gene ontology enrichment analyses were performed using the gwasGOenrich function with the following parameters: ontologies for cellular components, molecular function, and biological process were examined (ontology = “CC”, “MF”, or “BP”), pruneTermsBySize = 8, pkgname.GO = “org.Hs.eg.db”, and topGOalgorithm = “classic”. Correction for multiple testing was accomplished using q-values.

### Yatsunenko *et al*. data comparison

16S rRNA amplicon data from Yatsunenko *et al*.[46] was downloaded from MG-RAST on 12/14/2012 and pre-processed as described previously (base quality trimming to classification) using the same procedures as for the Hutterite samples to ensure data comparability[18]. Similar normalization and filtering steps were used to process classified reads from the Yatsunenko populations: bacterial taxa that were detected in fewer than 75% of individuals per population were eliminated and the remaining bacterial taxa relative abundances were fit to a standard normal distribution across individuals using qqnorm in R. Sex effects were examined using a linear model (lm in R) for the USA and Venezuelan populations (the Malawi sample consisted of all females). Q-values were calculated within each population to control for multiple testing, with q ≤ 0.05 chosen as the significance threshold. Hypergeometric tests were performed to determine whether there was significant overlap in the taxa showing differential abundance by sex in each pair of populations.

## Results

### Correlations of relative taxa abundance with age and sex

**Fig 1:**
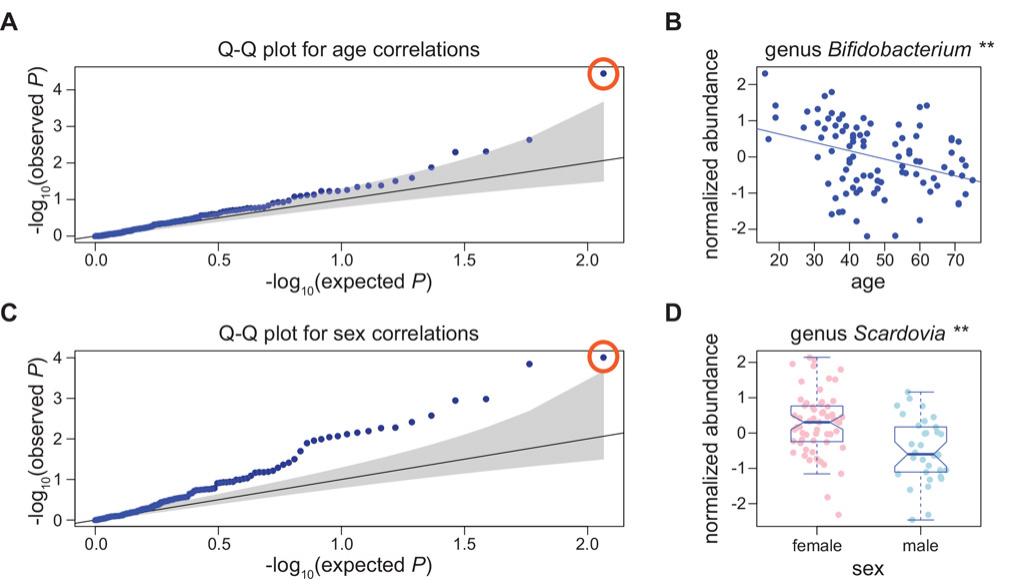
Bacterial abundance correlations with age and sex. A) Q-Q plot for correlations of 116 common bacterial taxa with age in samples collected in winter. Gray shading represents the 95% confidence interval of the null. The point circled in orange is genus *Bifidobacterium*. B) Abundance of genus *Bifidobacterium* is inversely correlated with age in samples collected during the winter (** q ≤ 0.01). C) Q-Q plot for correlations of 116 common bacterial taxa with sex in samples collected in winter. The point circled in orange represents genus *Scardovia*. D) Genus *Scardovia* was significantly more abundant in females (n = 60) than in males (n = 33) in winter (** q ≤ 0.01).

In a previous study of these individuals, we examined the relationship of microbiome alpha diversity to age and sex. While a significant association between diversity and sex was not observed in either winter or summer, diversity was significantly inversely correlated with age during the winter months (*P* < 0.01)[18]. Here, we sought to identify individual bacterial taxa whose relative abundances were correlated with sex or age in the three seasonal analyses (winter, summer, or “seasons combined”). At a q-value cutoff ≤ 0.05, the abundance of only one bacterial taxon was significantly inversely correlated with age in the winter samples (genus *Bifidobacterium*, Fig 1, Table 1, S5 Table). Members of *Bifidobacterium* have previously been shown to decrease in abundance with age in the gut[47, 48].

**Table 1:** Number of bacterial taxa that vary by sex or age in each season. The total number of taxa whose relative abundances were significantly correlated with age or sex at various q-value cutoffs are listed. Total number of taxa tested per season is indicated in the bottom row (total). In the text, we discuss the number of significantly correlated taxa in each season with a q-value threshold of ≤ 0.05. The abundances of few bacterial taxa appear to vary consistently with age; however, the abundances of many bacterial taxa are correlated with sex in this population.

	age			sex		
q-value cutoff	winter	summer	combined	winter	summer	combined
0.001	0	0	0	0	0	3
0.01	1	0	0	2	1	8
0.05^a^	1	0	0	4	5	18
0.1	1	1	2	16	19	24
0.2	4	5	2	19	30	49
Total examined	116	104	102	116	104	102

^a^ Confidence threshold chosen for significance

In contrast, many bacterial taxa showed sex specific abundance patterns (4/116 –winter, 5/104 –summer, 18/102 –“seasons combined”, Fig 1, Table 1, S6 Table). Moreover, bacterial taxa with sex-specific abundance patterns in multiple seasons tend to show the same direction of sex-specific effects, demonstrating that these are likely consistent sex differences over time (S2 Fig).

### Estimates of chip heritability

To examine the role that common host genetic variation might play in determining microbial abundance in the gut, we examined “chip heritability”. This measurement, often referred to as the proportion of variance explained (PVE), is the maximum amount of the variance in a phenotype that can be explained by the genetic variation interrogated in a GWAS framework[49]. This method has proven successful in studies addressing questions of missing heritability for traits such as height[49], working memory performance[50], and liver enzyme levels[51].

**Fig 2:**
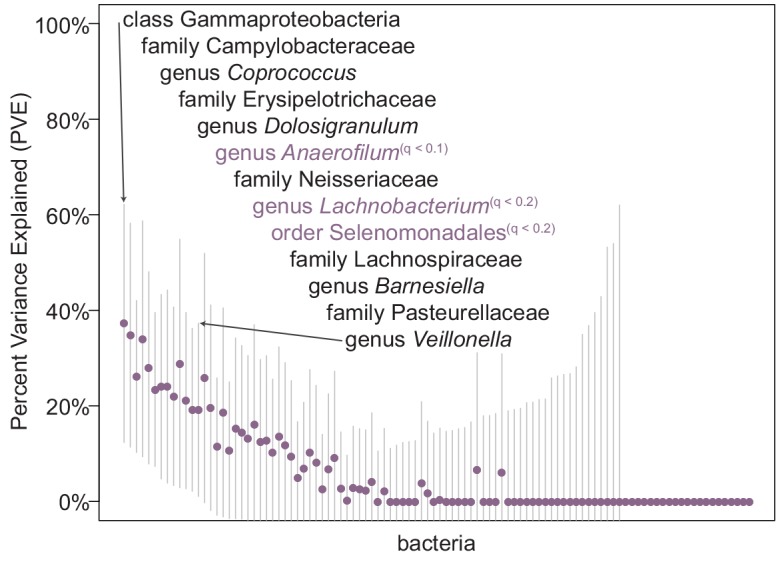
“Chip heritability” for 102 bacterial taxa tested in the “seasons combined” analysis. Each point represents the estimated percent variance explained (PVE, or “chip heritability”) for the joint effect of all genotypes analyzed in the GWAS for bacterial abundance during the “seasons combined” analyses. Bars indicate standard error measurements around the estimate. A number of bacterial taxa showed non-zero PVE estimates (listed in order from highest to lowest PVE) with error bars that do not intersect zero, indicating that cumulative common genetic variation can explain some portion of the variation in bacterial abundance observed between individuals. Bacterial taxa that also had at least one nominally significant genetic association at a genome-wide association level are labeled in purple, with the level of significance indicated (q ≤ 0.2 or q ≤ 0.1).

We calculated PVE for each bacterial taxon using ~200k SNPs, as implemented in GEMMA (see Materials and Methods). Approximately 10–13% of bacterial taxa in each season have non-zero “chip heritability” estimates, suggesting that a portion of the microbiome is heritable (winter: 14/116 taxa, summer: 10/104 taxa, “seasons combined”: 13/102 taxa; Fig 2, S2 Table, S3 Fig). The standard errors of “chip heritability” estimates are very large, so we have not drawn conclusions about the actual heritability estimate or compared estimates between taxa due to the uncertainty in the measurement, but conservatively consider estimates whose error bars do not intersect zero as showing evidence of heritability.

In addition to bacterial relative abundance, we examined the “chip heritability” for three alpha diversity metrics. Diversity measures were calculated at the genus level (richness—S, Shannon diversity—H, and evenness—J) and included all classified bacterial genera (see Materials and Methods). There was no evidence of “chip heritability” for any diversity metric in summer; however, evenness in the “seasons combined” analysis (“chip heritability”: J = 0.58 ± 0.32) and both evenness and Shannon diversity in winter (“chip heritability”: J = 0.56 ± 0.22, H = 0.52 ± 0.22) have non-zero estimates (S2 Table).

### Genome-wide association studies (GWAS)

To determine if specific variants in the human genome are associated with microbial abundance in the gut, we employed a classic GWAS approach for quantitative trait mapping. A large number of SNP-taxon associations were tested (~100 bacterial taxa + 3 diversity metrics per season x 3 seasons x ~200k SNPs per GWAS), which sets a study-wise Bonferroni corrected *P*-value threshold at 7.0 x 10^−10^. Given our sample size (~100 individuals per season), only associations with very large effect sizes would be expected to pass this significance threshold. Unsurprisingly, no variants passed this threshold in any of our analyses; therefore, we consider the individual SNP/taxon associations identified in these studies to be suggestive and requiring further replication.

Given this caveat, we identified genome-wide significant associations after correcting for multiple testing *within* each bacterial abundance GWAS individually, either by Bonferroni correction or by q-value (considering significance thresholds at both q ≤ 0.1 and q ≤ 0.2 cutoffs). Limiting the correction for multiple testing to the number of tests done within an individual GWAS is similar to the typical correction applied to GWAS with human disease (where the correction factor is determined by the parameters of a GWAS for a specific disease, not across GWAS in studies that consider multiple phenotypes[52–55]).

**Fig 3:**
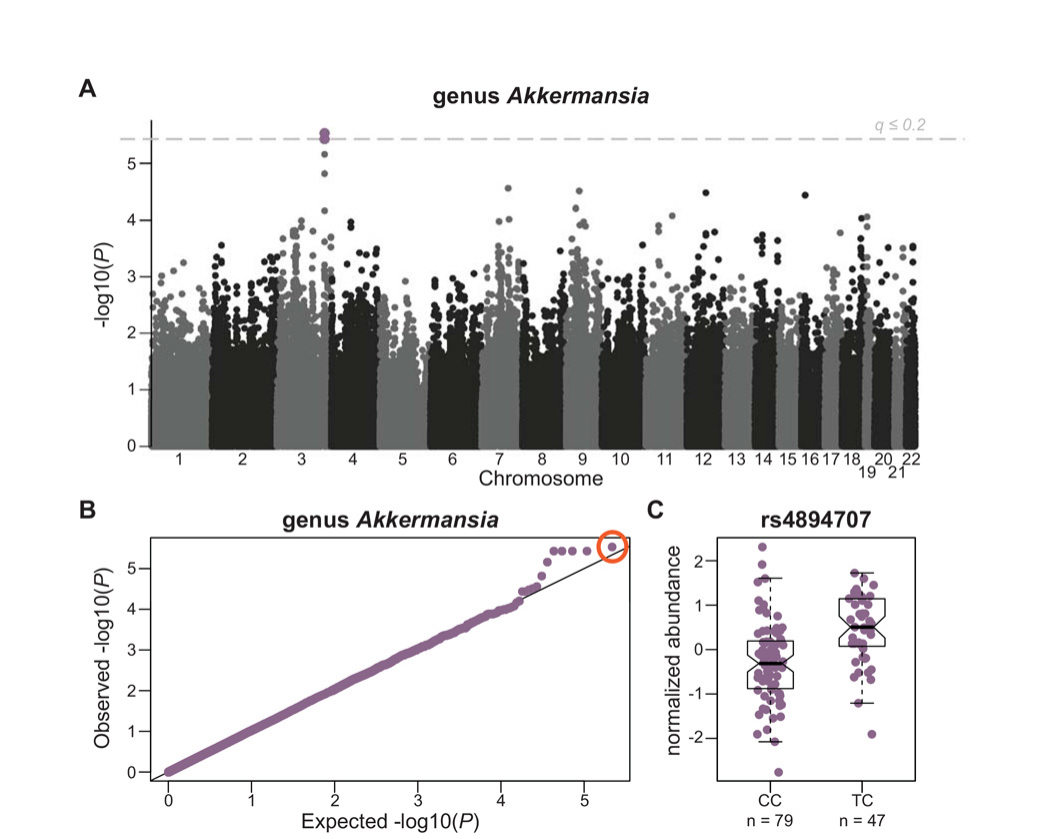
GWAS of genus *Akkermansia* relative abundance. A) Manhattan plot of GWAS results for the normalized relative abundance of genus *Akkermansia* from the “seasons combined” analysis. Each point represents a tested SNP, displayed by chromosomal position (x-axis). The y-axis shows–log_10_(*P*-value) for each SNP. SNPs significantly associated with normalized *Akkermansia* relative abundance (q ≤ 0.2) are shown in purple on chromosome 3. B) Q-Q plot for *P*-values from the GWAS of the relative abundance of genus *Akkermansia*. The majority of SNPs lie along the null line, demonstrating the test statistics did not appear to be inflated (due to population stratification, for example). Five SNPs (all in linkage disequilibrium (LD) on chromosome 3) were significantly associated with *Akkermansia* abundance. The point circled in orange was the most highly associated SNP (rs4894707). C) Normalized *Akkermansia* abundance, segregated by genotype class at rs4894707 on chromosome 3. Only two genotype classes are represented at this SNP (MAF = 0.185 and Hardy-Weinberg *P*-value = 0.007 in a larger sample of 1,415 Hutterites that includes the individuals in this study). This SNP lies in a UTR region of the gene *PLD1*, which as been implicated in obesity studies in African American populations[56].

At least one bacterial taxon per season was significantly associated with at least one SNP (at a Bonferroni significance threshold), and the abundances of at least eight bacterial taxa are associated with at least one SNP at q-values less than or equal to 0.2 (Table 2, S7 Table, Fig 3). There were no significant associations with alpha diversity metrics in any of the analyses.

**Table 2:** Number of bacterial taxa with at least one SNP association reaching suggestive significance per season. For each season, the total number of bacterial taxa examined and number of single-nucleotide polymorphisms (SNPs) tested are listed. The number of taxa for which at least one SNP was associated with abundance at either a Bonferroni corrected *P*-value cutoff or a q-value cutoff of 0.1 or 0.2 are listed. The total number of taxa for which at least one associated SNP fell below q-value ≤ 0.2 or a Bonferroni threshold are listed under “total significant”.

	Total		Thresholds of significance			
Season	Bacterial taxa	SNPs	Bonferroni	q ≤ 0.1	q ≤ 0.2	Total significant
Winter	116	211,319	2	8	15	15
Summer	104	210,924	1	6	14	14
Combined	102	212,153	1	2	8	8

We compared the results of our genome-wide association analyses to the “chip heritability” estimates for each bacterial taxon and identified bacterial taxa that have both significant “chip heritability” and at least one suggestive genome-wide significant association (winter: 6 taxa, summer: 2 taxa, “seasons combined”: 3 taxa, Fig 2, see Materials and Methods). Overall, taxa with non-zero “chip heritability” for a given season were significantly enriched for having at least one genome-wide significant SNP association using the winter microbiome data (permutation *P* < 0.01), were slightly enriched for significant associations in the analysis of data from both seasons (*P* = 0.07), but were not enriched for genome-wide associations in the summer microbiome data (*P* = 0.4, S4 Fig).

### Gene set enrichment analysis (GSEA)

In order to gain insight into the type of host pathways and functions that might influence bacterial abundance in the gut, we performed gene set enrichment analysis (GSEA) on all GWAS with at least one genome-wide significant association in our study (S2 File, see Materials and Methods). For most bacterial GWAS, we do not observe enrichment of any gene ontology (GO) categories given the distribution of *P*-values of SNPs located in or near annotated genes in the genome (within 10kb). However, for several bacterial GWAS (winter: 4 taxa, summer: 10 taxa, “seasons combined”: 6 taxa), GSEA resulted in significant enrichments for biological processes one might expect to be interacting with the microbiome (q ≤ 0.2). For example, GSEA revealed enrichments of genes categorized under immune processes (summer: genus *Sporacetigenium*) and metabolic processes (winter: order Burkholderiales; summer: genus *Sporacetigenium*; “seasons combined”: genus *Megasphaera*), as well as in pathways that generate ribosomal components (summer: genus *Anaerostipes*; “seasons combined”: genus *Anaerofilum*) and act in multi-organism process and communication (“seasons combined”: genus *Anaerostipes*). Finally, enrichment in olfactory receptor pathways was observed for a number of taxa (winter: family Succinivibrionaceae; summer: genus *Bifidobacterium*, order Rhizobiales; “seasons combined”: genus *Anaerofilum*, genus *Faecalibacterium*). One caveat to note is that olfactory receptor genes tend to be clustered in the genome, which can lead to spurious enrichment in gene ontology tests.

### Identification of candidate tissues

Our GWAS revealed a number of candidate variants that potentially influence gut microbiome composition; however, we lack an understanding of the relevant tissues in which these variants act. We sought to provide insight into this question by intersecting our GWAS results with DNase hypersensitivity (DHS) data, following the approach of Maurano *et al*.[43]. Cell types identified through their analysis were deemed “pathogenic cell types”; here we will refer to them simply as candidate tissues because the unknown relationships of our GWAS results to disease.

We performed this candidate tissue identification analysis for each of our GWAS of bacterial abundance when either i) at least one SNP met suggestive genome-wide significance, or ii) there was non-zero “chip heritability” for that taxon (number of taxa examined in winter = 23, summer = 22, “seasons combined” = 18). When considering all bacterial taxa that met our inclusion criteria from either the winter or summer data sets, we do not observe more candidate tissues identified at nominal significance than we would expect by chance (S5 Fig). In contrast, for taxa meeting our inclusion criteria from the “seasons combined” analysis, we observed more candidate tissues of nominal significance than would be expected by chance; therefore we only considered results from the “seasons combined” analysis further.

**Fig 4:**
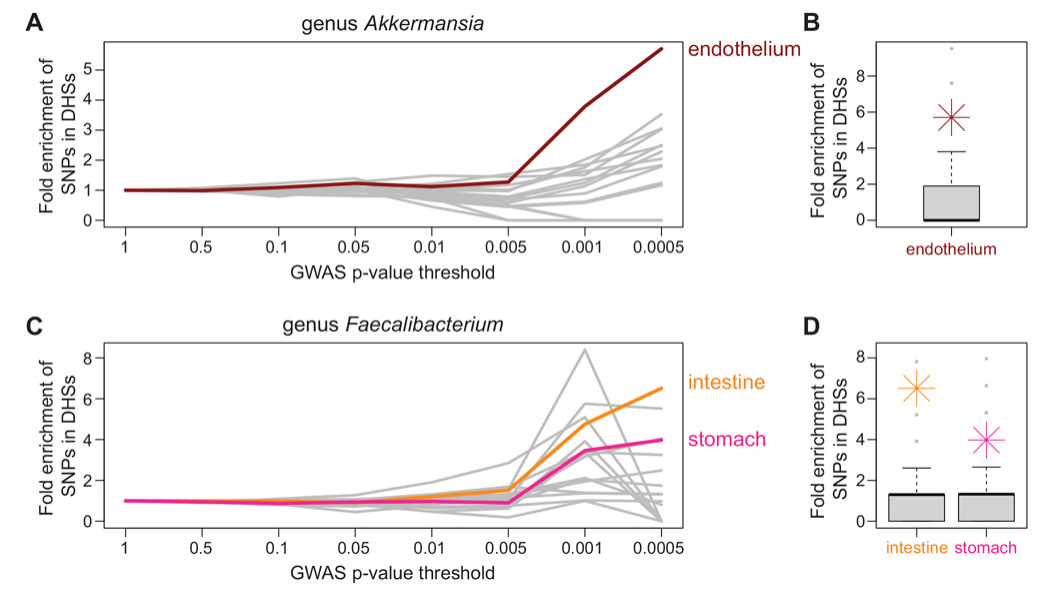
Identification of candidate tissues. At increasingly significant *P*-value thresholds, variants identified through GWAS were enriched in DNase hypersensitivity peaks in a tissue-specific manner. A) For genus *Akkermansia*, low *P*-value GWAS SNPs were significantly enriched in DHS peaks in endothelial cell types (red), but not in DHS peaks of the 15 other tissues examined (gray). The x-axis shows the *P*-value threshold bins examined and y-axis represents fold enrichment for SNPs overlapping DHS peaks in that bin compared to genome-wide for that tissue type. Both the abundance of genus *Akkermansia* and endothelial barrier function have been associated with obesity, providing a mechanistic hypothesis that can be further investigated. B) For genus *Akkermansia*, the significance of enrichment of GWAS SNPs overlapping DHS peaks in endothelial tissue in the lowest *P*-value bin (*P* ≤ 0.0005) was determined by GWAS permutation (*P* ≤ 0.05, see Materials and Methods). The distribution of permuted GWAS SNP enrichments in DHS peaks of endothelial tissue is displayed as a boxplot with actual enrichment plotted as red star. C) For genus *Faecalibacterium*, low *P*-value GWAS SNPs are significantly enriched in DHS peaks of both intestine (orange) and stomach (pink) tissues (*P* ≤ 0.05). D) For genus *Faecalibacterium*, the significance of enrichment of GWAS SNPS overlapping DHS peaks of both intestine and stomach tissues in the lowest *P*-value bin (*P* ≤ 0.0005) was determined by GWAS permutation (intestine *P* ≤ 0.01, stomach *P* ≤ 0.05, see Materials and Methods). Members of *Faecalibacterium* are some of the most common species in the gut and are known to be associated with dysbiosis in patients with irritable bowel syndrome. The distribution of permuted enrichments for each identified candidate tissue is displayed as a boxplot with actual enrichment plotted as an orange (intestine) and pink (stomach) star.

For five bacterial taxa we are able to identify at least one candidate tissue (out of 18 total taxa that met inclusion criteria from “seasons combined” analyses). Endothelial tissues were identified for genus *Akkermansia* and muscle tissues for genus *Dolosigranulum*. Both intestine and stomach tissues were identified as potential candidates for genus *Faecalibacterium*. Family Neisseriaceae and family Pasteurellaceae both have a large number of tissues identified as candidates (Fig 4, S6 Fig).

## Discussion

### The role of host genetics in gut microbiome composition

In this study, we examined the role of host genetics in determining gut microbiome composition in an isolated, communally living population: the Hutterites. We first explored non-genetic effects of age and sex, and demonstrated that at least four bacterial taxa show sex specific patterns of abundance in each season. We further demonstrated that at least 13 bacterial taxa show evidence of heritability in each season and ultimately identified at least eight bacterial taxa in each season whose abundances were significantly associated with genetic variation in the human genome. Finally, we examined host tissue types in which genetic variation may be acting to influence gut microbial composition.

Although no human replication cohorts are published, we replicated a previously reported association from a quantitative trait locus (QTL) mapping study of microbial abundance in the gut of advanced intercross mouse strains[30]. In that study, genus *Lactococcus* showed strong evidence of association (LOD score = 8) with markers on mouse chromosome 10. In our study, genus *Lactococcus* also showed evidence of genetic association in the summer (rs3747113, *P* = 3.13 x 10^−7^). Interestingly, the associated variant in the Hutterites is located in the syntenic linkage interval observed in the mouse QTL study, providing evidence of replication of this association across species. The size of the linkage interval in Benson *et al*. spans tens of megabases of the mouse genome, however, and finer mapping is needed to confirm replication.

One of the more biologically interesting results of our study was the identification of SNPs that were associated with abundance levels of genus *Akkermansia* (Fig 3). These SNPs lie within intronic and untranslated regions (UTR) of the gene *PLD1*, which is thought to play a role in signal transduction and subcellular trafficking[57–59]. This gene was implicated in a previous GWAS of body mass index (BMI) in African Americans, because a SNP associated with BMI is located near the gene[56]. In addition, increased abundance of *Akkermansia muciniphila* was recently shown to be protective against developing obesity in mice[60]. These correlations between a bacterial taxon, genetic variation, and BMI possibly suggest a mechanism for how genetic variation in or near this gene acts to influence obesity. Further support for this mechanism was provided by the *de novo* candidate tissue identification in which SNPs with low *P*-values from the genus *Akkermansia* GWAS were significantly enriched in DHS peaks of endothelial cell types (Fig 4). Endothelial barrier function is thought to be important in obesity. For example, in swine fed with a high-fat diet to promote obesity, endothelial barriers became more permeable early during weight gain[61]. In addition, endothelial permeability is regulated by the immune system[62–64], and there are strong links between alterations in the immune system with obesity[65–67]. We did not observe associations between BMI and the relative abundance of genus *Akkermansia* in the Hutterite sample (*P* = 0.51); but BMI was measured on these individuals 3–5 years prior to microbiome sampling. Further investigation is needed to validate this intriguing hypothesis by confirming the relationship of *Akkermansia* to *PLD1* and obesity, ideally within a set of samples where microbiome and obesity measures are collected concurrently.

### Host cell types and pathways implicated from GWAS results

In addition to examining human variation that met statistical thresholds at a genome-wide significance level, gene-set enrichment analysis revealed a number of human cellular pathways that might be an important interface between the host and the microbiome. In particular, olfactory receptor activity was significant in GSEA for five taxon GWAS (winter: family Succinivibrionaceae; summer: genus *Bifidobacterium*, order Rhizobiales; combined: genus *Anaerofilum*, genus *Faecalibacterium*). It has already been demonstrated that olfactory receptors form an interface between the host and the gut microbiota. In mice, an olfactory receptor expressed in the kidneys responds to metabolites produced by gut bacteria, and this process aids in regulating blood pressure systemically via renin production[68]. It is possible that additional olfactory receptors in other tissues may also recognize compounds produced by the microbiota and act as a way for the host to regulate either host physiology or the microbiome in response to the gut environment. These results demonstrate that host genetic variation may exert control over microbial abundance through a variety of mechanisms, such as through immune system interaction, metabolism, energy availability, and potentially olfactory receptor activity.

Genetic variation in identified pathways could be acting in a number of host tissues to influence bacterial composition in the gut, either directly (for example, hormones produced in the brain influence bacteria in the gut[69]) or indirectly (for example, microbial byproducts processed by the liver[70]). For the genus *Faecalibacterium*, both stomach and intestines are identified as candidate tissues where host genetic variation may be acting to influence bacterial abundance (Fig 4). *F*. *prausnitzii*, *a* species of *Faecalibacterium*, is one of the most common gut bacteria in adults and has been well characterized[71]. This bacterium lives along the mucus interface in the gut[72] and is known to affect expression of host mucus glycans[73]. High levels of *F*. *prauznitzii* are thought to be protective against ulcerative colitis[74], Crohn’s disease[75–77], and celiac disease[78]. Given the roles that members of this taxon play in gut health, it is interesting to observe DHS peaks in stomach and intestinal tissues are significantly enriched for the most strongly associated GWAS variants. Considered together, these results provide insight into how host genetic variation may be acting to influence bacterial abundance in the gut.

### The role of sex in gut microbiome composition

In addition to examining the role of host genetics in determining gut microbiome composition, we also identified bacterial taxa that are differentially abundant by sex. In the Hutterites, at least four bacterial taxa differ in abundance between the sexes each season, including genus *Scardovia*, genus *Gordonibacter*, genus *Anaerotruncus*, and phylum Proteobacteria. These abundance differences are directionally consistent across season and there are a number of hypotheses for these observations. One potential explaination is that inherent biological differences between the sexes (for instance hormone levels) could drive the observed bacterial abundance differences. Alternatively, the division of labor could drive sex specific differences between men and women in Hutterite society[79]. For example, Hutterite men typically work in the income-generating jobs, which vary by colony. Younger men might work in the fields, barns, or machine shops, while older men take on positions of leadership in the colonies. In contrast, Hutterite women perform family, domestic, and food preparation jobs, including cooking, cleaning, gardening, and sewing. It is possible that men and women are exposed to different environmental microbes due to differences in their daily activities. A similar notion was suggested previously in a study of Hadza hunter-gatherers, where sex differences in the relative abundances of three taxa of the gut microbiome were observed[80]. The authors attributed those differences to the division of labor between men and women in that society (men tend to forage further from camp and for different food sources than women, who remain near to camp to stay with the children).

To further investigate these two hypotheses, we examined whether sex is associated with bacterial abundances in additional populations, including individuals from the USA and Venezuela, using the data produced by Yatsunenko *et al*.[46] (see Materials and Methods). If underlying physiological differences between the sexes leads to varied bacterial compositions, we might expect to observe sexual dimorphism in the abundance of the same bacterial taxa across different geographies and cultures. While 14 bacterial taxa showed significant differential abundance by sex in the Yatsunenko USA population, none were significantly differentially abundant after multiple testing corrections in the Venezuelan populations (S8 Table, S7 Fig). Additionally, there was no overlap between the taxa identified as differentially abundant by sex in the Hutterites or in the Yatsunenko USA population (S9 Table). Given the differences in microbial exposures and in the uniformity of the environments within the Yatsunenko and Hutterite populations, it is unclear whether sex differences in the microbiome are due to biological or cultural factors, or a combination of both.

In our previous study of seasonal effects on the gut microbiome in the Hutterites[18], we did not observe differences in alpha diversity metrics (Shannon diversity, species richness, or species evenness) between men and women in either winter or summer. Conversely, when we considered individual bacterial taxon abundances we observed many sex differences in this population. These results highlight an important phenomenon that should be considered in microbiome studies: trends observed in broad summary statistics, such as diversity, are not always observed at individual taxon levels and vice versa.

### Limitations and conclusions

Although the results of our study indicate that host genetics plays a role in determining gut microbiome composition, there are important limitations. The first is the relatively small size of our sample (n ≈ 100 individuals in each season). For many GWAS of common diseases, the sample sizes necessary to detect significant associations are thousands to tens of thousands of individuals. We proposed that the relatively uniform environment that the Hutterites are exposed to, in particular their communal diet, would reduce variation due to non-genetic factors and increase our power to detect genetic associations. While this may be true, it is clear that much larger sample sizes will be needed to ensure sufficient power to detect association with the high multiple testing burden and for narrowing the confidence intervals on heritability estimates. A second limitation is that published replication cohorts are not currently available for these traits in humans and we are unable to confirm any associations we detect in independent human samples.

Despite these limitations, we do replicate a previously observed bacterial abundance QTL in mouse[30]. Additionally, the candidate tissue identification analysis provided additional validation of our results, as we would not expect any kind of enrichment if our results were all spurious. Finally, several lines of evidence point towards a relationship of genus *Akkermansia*, in particular, to host genetics, including genome-wide significant GWAS hits (Fig 3), endothelial cell type enrichments for the top associations (Fig 4), and biological plausibility[56, 60]. Given the medical importance of this genus[60, 81], further work should be performed to confirm this relationship and further explore how host genetics might influence this taxon.

This study is one of the first to explore host genetic influences on gut microbiome composition on a genome-wide scale in humans. We identified bacterial taxa that show sex specific patterns of abundance in the Hutterites, although it is unclear whether biological or cultural factors are driving these patterns. We identified at least 10 bacterial taxa in each season that appear to be heritable, by examining “chip heritability”. At least seven bacterial taxa in each season are associated with variation in the human genome at a genome-wide significance level when considering the number of SNP tested within each GWAS. Gene set enrichment analysis demonstrated that the SNPs identified in these GWAS likely function through a variety of different mechanisms in the body including immune function, metabolism, and energy regulation. Finally, candidate tissues where host genetic variation might act to influence microbial abundance in the gut were identified. This work offers a first glimpse into the role human genetics plays in maintaining gut microbiome composition.

## Supporting Information

S1 Fig Principal components analysis (PCA) of “seasons combined” analysis. PCA was performed on all individuals (genus level data) after combining data that had been normalized within season first. Quantile normalization within each season separately before combining data eliminates seasonal differences along the top 10 principal components (PCs 1 and 2 plotted here, linear model *P* > 0.05).
(TIFF)

S2 Fig Bacterial taxa that show correlations with sex in at least two seasons examined. Each of these bacterial taxa shows differential abundance by sex in at least two seasons examined. For most bacterial taxa, the direction of association stays constant between males and females regardless of season, pointing to consistent variation of bacterial abundance between the sexes. Significance: q ≤ 0.05 = *, q ≤ 0.01 = **, q ≤ 0.001 = ***, ns = not significant). Bacterial taxa include A) genus *Clostridium*, B) genus *Collinsella*, C) genus *Gordonibacter*, D) genus *Mitsuokella*, E) genus *Scardovia*, F) order Burkholderiales, G) phylum TM7.
(TIFF)

S3 Fig “Chip heritability” across season. “Chip heritability” (or Percent Variance Explained, PVE, circle) along with the standard error around the estimate (gray bars) is shown for the bacterial taxa observed in all three seasonal analyses. Bacterial taxa are ordered by the PVE in the “seasons combined” analysis. Filled circles indicate taxa that had at least one genome-wide significant SNP association in that season (either q ≤ 0.2 or Bonferroni corrected). Open circles indicate taxa that did not have any SNPs associated with their abundance in that season after correcting for multiple tests genome-wide.
(TIFF)

S4 Fig Overlap of bacterial taxa showing evidence of heritability and GWAS signals. The number of overlaps between bacterial taxa showing heritability (from “chip heritability” estimation) and bacterial taxa with at least one genome-wide significant association from GWAS is shown for each season with the colored, dashed line. A null distribution of overlaps expected by chance given the number of bacterial taxa showing heritability and showing a GWAS hit was calculated by permuting which bacterial taxa were labeled as heritable 1000 times. An empirical p-value was calculated by dividing the number of overlaps observed in the permutations greater than the actual overlap by the total number of permutations (1000). This was done for each season: A) winter, B) summer, and C) “seasons combined”.
(TIFF)

S5 Fig Distribution of p-values from candidate tissue identification analyses. *P*-values were calculated via permutation for each tissue for each bacterial taxon that had either non-zero “chip heritability” or at least one genome-wide significant SNP. The number of expected associations with a *P* ≤ 0.05 is indicated with the red line on each plot. As can be seen from the histograms, most tissues do not show enrichment for low GWAS p-value SNPs in DHS peaks (large excess around *P* = 1). For winter (A) and summer (B), there are not more associations at *P* ≤ 0.05 than we would expect by chance. However, in the “seasons combined” analysis (C), there is a slight excess of *P*-values ≤ 0.05, indicating significant associations exist in this analysis (although many false positives likely exist as well). Therefore, candidate tissue analysis for winter and summer were not considered further.
(TIFF)

S6 Fig Additional candidate tissues identified in “seasons combined” analysis. A, C, and E) GWAS variants with increasingly low *P*-values (x-axis in (A), (C), and (E)) are enriched (y-axis) in DNase Hypersensitivity Sites (DHS) for various tissues, compared to the genome-wide distribution of GWAS SNPs falling in DHS peaks for that tissue. Identified tissues are listed in the top left corner for each bacterial taxon examined (color matches lines on plot and order is the same as order in the y-dimension in the lowest *P*-value bin). Any tissue not listed (drawn in the thin, gray line) did not have significant enrichment in the lowest *P*-value bin. B, D, and F) Boxplots (gray) indicate the null distribution of enrichments of SNPs in the lowest GWAS *P*-value bin in DHS peaks for the given tissues calculated from GWAS permutations from (A), (C), and (E), respectively. The actual enrichment of SNPs in the lowest GWAS *P*-value bin overlapping DHS peaks for each tissue is indicated by the colored star. All enrichments shown have a *P* ≤ 0.05. A & B) family Neisseriaceae, C & D) family Pasteurellaceae, and E & F) genus *Dolosigranulum*.
(TIFF)

S7 Fig Distribution of p-values for sex correlations in Yatsunenko populations. A and C) Histograms of the *P*-values for bacterial taxa correlated with sex in the USA (A) and Venezuelan (C) populations. B and D) Quantile-quantile plots (Q-Q plots) of the -log_10_(*P*-values) for sex correlation testing in the USA (B) and Venezuelan (D) populations. In the USA, there is an excess of low *P*-values, indicating many bacterial taxa show differential abundance by sex.
(TIFF)

S1 File GWAS SNP enrichments in tissue DHS. This file directory contains three folders, one for each seasonal analysis (winter, summer, “seasons combined”). Within each folder there is one text file per bacteria tested. Each text file contains 17 rows and variable numbers of columns. Each row represents one tissue type as determined by clustering analysis of DHS peaks (along with a header). Each column represents a GWAS p-value threshold examined. The minimum threshold chosen for each bacterium contains at least 50 SNPs. Entries are the enrichment of GWAS SNPs under that threshold compared to genome-wide for that tissue type.
(ZIP)

S2 File Gene set enrichment analysis (GSEA) results. This folder contains GSEA results for all that had at least one genome-wide significant hit (either after Bonferroni correction or q < 0.2). It contains three folders (one per seasonal analysis–winter, summer, “seasons combined”). Each folder contains three files per bacterial taxa tested (categories from GO: BP—"biological process", CC—"cellular compartment", MF—"molecular function"). Only GO categories with a q < 0.2 were examined. If a file contains only header information, there were no significant enrichments.
(ZIP)

S1 Table Taxa filtered out during QC. This table contains the list of bacteria that were trimmed due to high correlation with another bacterium for the "winter", "summer", and "seasons combined" analyses.
(XLSX)

S2 Table "Chip heritability" for each bacterial taxon. This table contains proportion of variance explained (PVE) estimates ("chip" heritability) for the "winter", "summer", and "seasons combined" analyses and reported by GEMMA.
(XLSX)

S3 Table Tissue classification for enrichment analysis. This table lists the DNase Hypersensitivity Site (DHS) sample names from Maurano *et al*. and the tissue it was classified as using the clustering procedure described in the methods.
(XLSX)

S4 Table Candidate tissue p-values. This table contains *P*-values representing the significance of enrichment of GWAS SNPs overlapping DHS peaks in each of the 16 tissues examined in the lowest GWAS p-value bin tested for each bacterial taxon.
(XLSX)

S5 Table Associations of bacterial taxa to age. This table contains tables of *P*-values and q-values for age correlations done in GEMMA for the "winter", "summer", and "seasons combined" analyses.
(XLSX)

S6 Table Correlations of bacterial taxa to sex. This table contains tables of *P*-values and q-values for sex correlations done in GEMMA for the "winter", "summer", and "seasons combined" analyses.
(XLSX)

S7 Table Top GWAS hits. This table contains all SNPs that met a genome-wide significance threshold either with a *P*-value after Bonferroni correction or with a q-value < = 0.2.
(XLSX)

S8 Table Associations of bacterial taxa to sex in Yatsunenko populations. This table contains tables of *P*-values and q-values for the sex associations (linear model, see methods) for the USA and Venezuelan individuals from Yatsunenko et al. A q-value < = 0.05 was considered significantly differentially abundant between the sexes.
(XLSX)

S9 Table Overlap of differentially abundant bacterial taxa by sex in the Hutterite and Yatsunenko populations. This table lists the number of bacterial taxa that showed evidence of being differentially abundant by sex in all three Hutterite seasons examined (winter, summer, and “seasons combined”) and two of the Yatsunenko populations (USA and Venezuela). Only the taxa identified as common in both populations examined were considered. The significance of the overlap in bacterial taxa identified in each population is listed (see methods).
(XLSX)
