# Supplementary figures and images for: Genome-Wide Association Studies of the Human Gut Microbiota
Source: PLoS One. 2015 Nov 3;10(11):e0140301. doi: 10.1371/journal.pone.0140301 (PMC4631601; doi:10.1371/journal.pone.0140301)

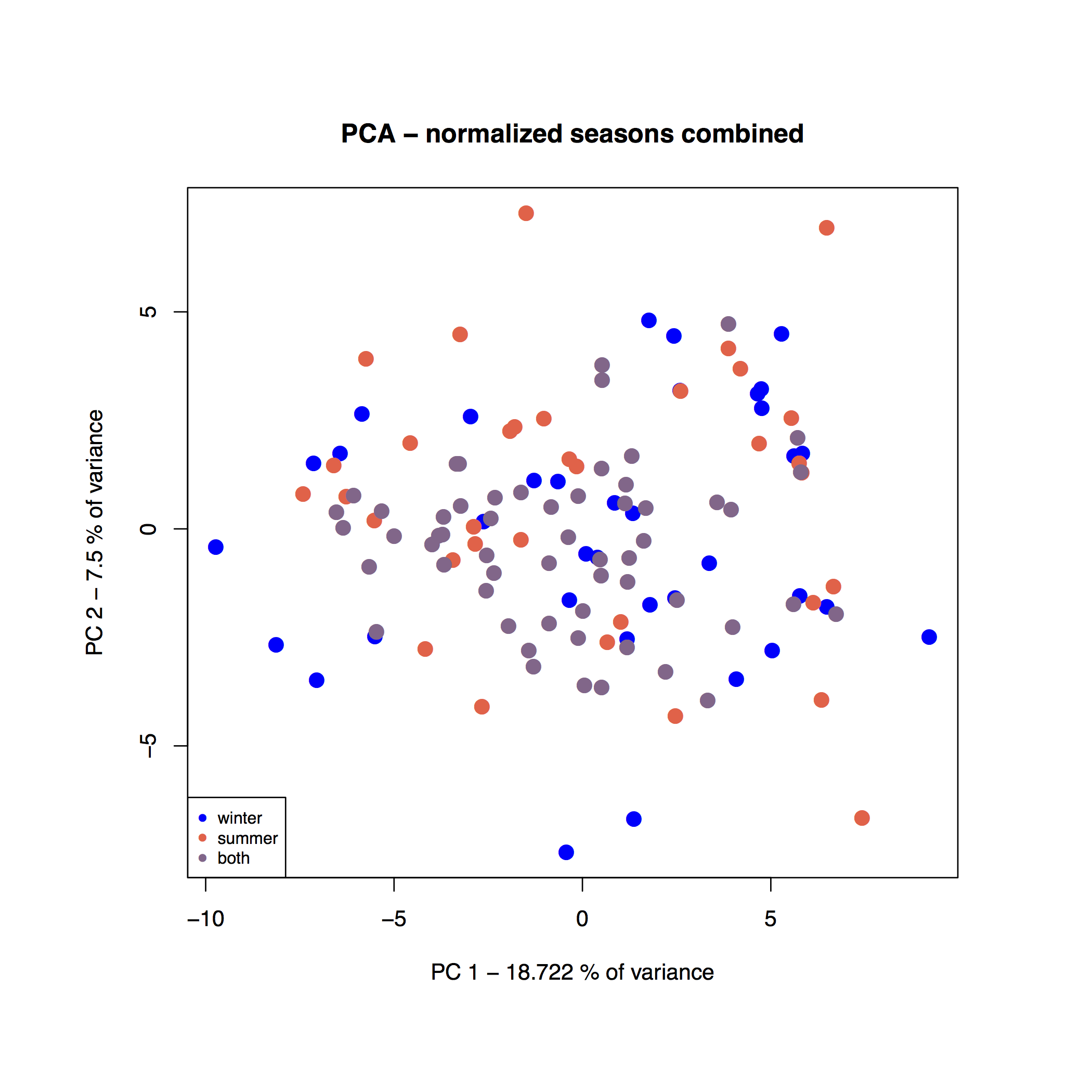

Supplement: S1 Fig — PCA was performed on all individuals (genus level data) after combining data that had been normalized within season first. Quantile normalization within each season separately before combining data eliminates seasonal differences along the top 10 principal components (PCs 1 and 2 plotted here, linear model P > 0.05). (TIFF) [file pone.0140301.s001.tiff]

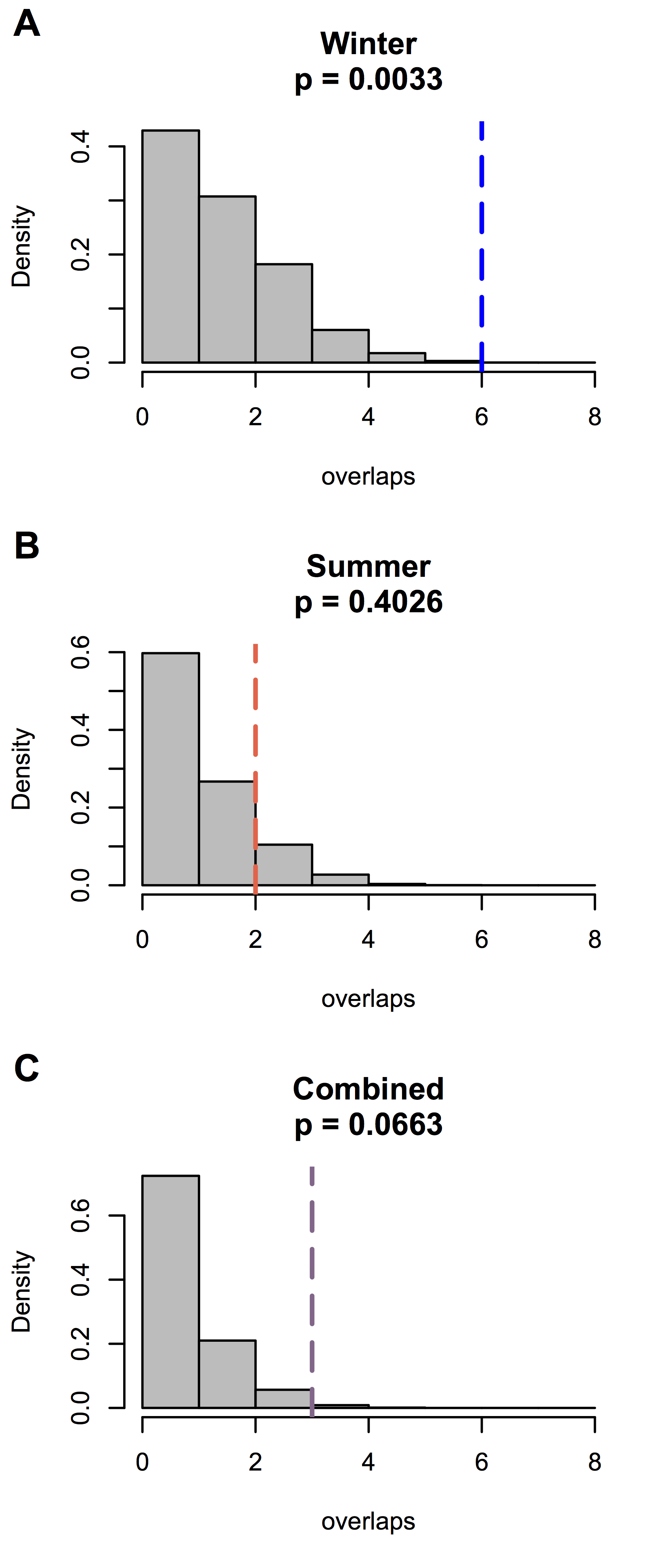

Supplement: S4 Fig — The number of overlaps between bacterial taxa showing heritability (from “chip heritability” estimation) and bacterial taxa with at least one genome-wide significant association from GWAS is shown for each season with the colored, dashed line. A null distribution of overlaps expected by chance given the number of bacterial taxa showing heritability and showing a GWAS hit was calculated by permuting which bacterial taxa were labeled as heritable 1000 times. An empirical p-value was calculated by dividing the number of overlaps observed in the permutations greater than the actual overlap by the total number of permutations (1000). This was done for each season: A) winter, B) summer, and C) “seasons combined”. (TIFF) [file pone.0140301.s004.tiff]

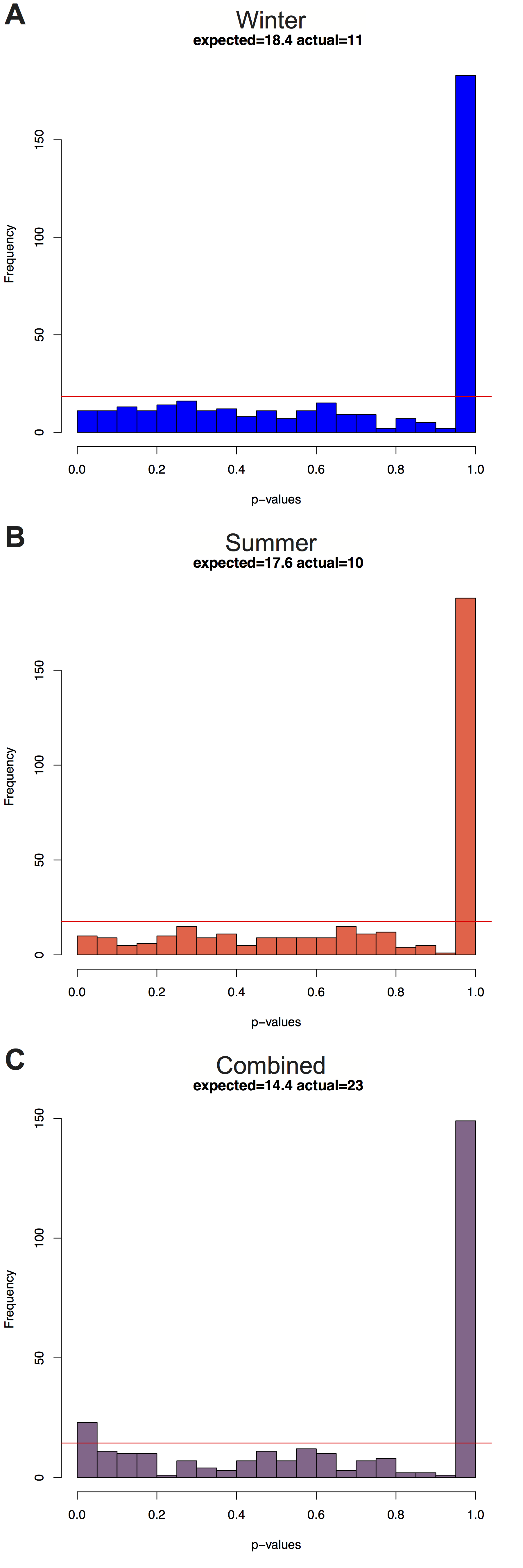

Supplement: S5 Fig — P-values were calculated via permutation for each tissue for each bacterial taxon that had either non-zero “chip heritability” or at least one genome-wide significant SNP. The number of expected associations with a P ≤ 0.05 is indicated with the red line on each plot. As can be seen from the histograms, most tissues do not show enrichment for low GWAS p-value SNPs in DHS peaks (large excess around P = 1). For winter (A) and summer (B), there are not more associations at P ≤ 0.05 than we would expect by chance. However, in the “seasons combined” analysis (C), there is a slight excess of P-values ≤ 0.05, indicating significant associations exist in this analysis (although many false positives likely exist as well). Therefore, candidate tissue analysis for winter and summer were not considered further. (TIFF) [file pone.0140301.s005.tiff]

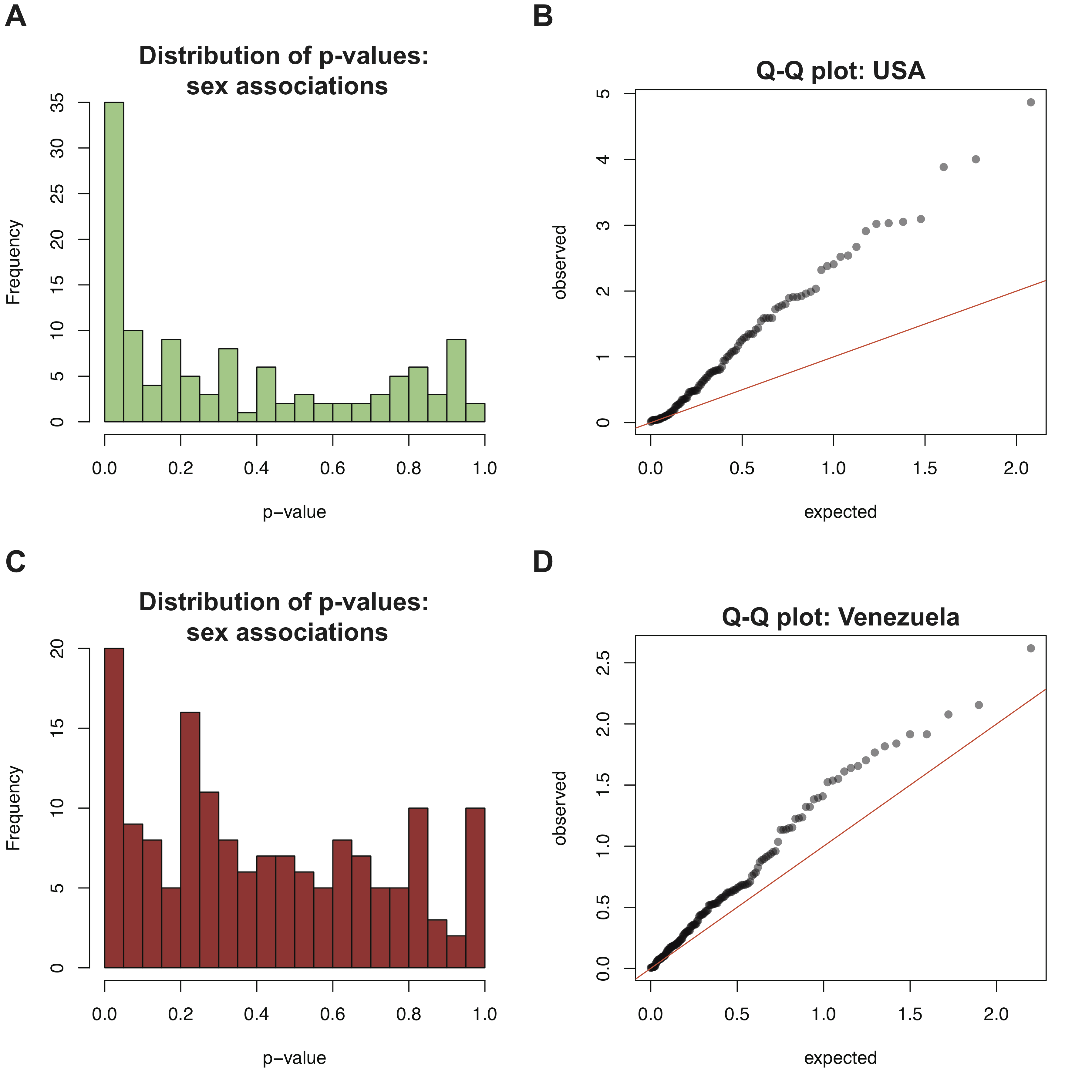

Supplement: S7 Fig — A and C) Histograms of the P-values for bacterial taxa correlated with sex in the USA (A) and Venezuelan (C) populations. B and D) Quantile-quantile plots (Q-Q plots) of the -log10(P-values) for sex correlation testing in the USA (B) and Venezuelan (D) populations. In the USA, there is an excess of low P-values, indicating many bacterial taxa show differential abundance by sex. (TIFF) [file pone.0140301.s007.tiff]
